# The Met1-linked ubiquitin machinery in inflammation and infection

**DOI:** 10.1038/s41418-020-00702-x

**Published:** 2021-01-20

**Authors:** Berthe Katrine Fiil, Mads Gyrd-Hansen

**Affiliations:** 1 LEO Foundation Skin Immunology Research Center, Department of Immunology and Microbiology, University of Copenhagen, Maersk Tower, Blegdamsvej 3B, DK-2200 Copenhagen, Denmark; 2 Ludwig Institute for Cancer Research, Nuffield Department of Clinical Medicine, University of Oxford, Old Road Campus Research Building, Oxford, OX3 7DQ UK

**Keywords:** Signal transduction, Antimicrobial responses, Cell death and immune response

## Abstract

Ubiquitination is an essential post-translational modification that regulates most cellular processes. The assembly of ubiquitin into polymeric chains by E3 ubiquitin ligases underlies the pleiotropic functions ubiquitin chains regulate. Ubiquitin chains assembled via the N-terminal methionine, termed Met1-linked ubiquitin chains or linear ubiquitin chains, have emerged as essential signalling scaffolds that regulate pro-inflammatory responses, anti-viral interferon responses, cell death and xenophagy of bacterial pathogens downstream of innate immune receptors. Met1-linked ubiquitin chains are exclusively assembled by the linear ubiquitin chain assembly complex, LUBAC, and are disassembled by the deubiquitinases OTULIN and CYLD. Genetic defects that perturb the regulation of Met1-linked ubiquitin chains causes severe immune-related disorders, illustrating their potent signalling capacity. Here, we review the current knowledge about the cellular machinery that conjugates, recognises, and disassembles Met1-linked ubiquitin chains, and discuss the function of this unique posttranslational modification in regulating inflammation, cell death and immunity to pathogens.

## Graphical abstract

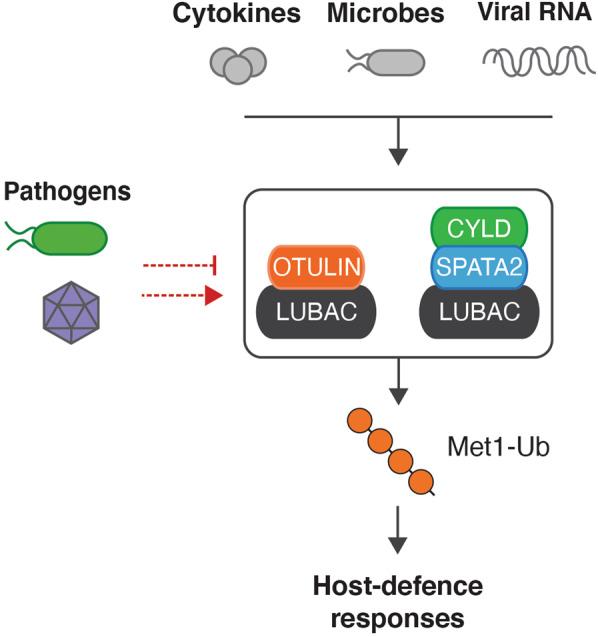

## Facts

- Met1-linked ubiquitin chains are key regulators of inflammation and immunity to pathogens.
- Met1-linked ubiquitin chains are assembled by LUBAC and are disassembled by the deubiquitinases OTULIN and CYLD.
- Met1-linked ubiquitin chains function as kinase scaffolds to control signalling outcomes by pattern recognition receptors and cytokine receptors.
- Intracellular bacteria are decorated with Met1-linked ubiquitin chains for activation of xenophagy.
- Pathogen-encoded effectors target the Met1-linked ubiquitin machinery to subvert host-defence responses.

## Open Questions

- What are the mechanisms that regulate the Met1-linked ubiquitin machinery?
- What is the function of individual LUBAC–deubiquitinase complexes?
- Can the Met1-linked ubiquitin machinery be targeted therapeutically to improve host-defence responses?

## Introduction

Ubiquitin (Ub) modification of proteins controls most cellular processes and is, alongside phosphorylation, the most widely used posttranslational modification (PTM) in cells [1]. Ub chains are well-known to target proteins for proteasomal degradation but non-degradative Ub chains have emerged as crucial signalling modules for innate immune responses [2]. These chains control inflammatory signalling, restriction of bacterial growth, and cell death triggered by innate immune receptors upon activation by infectious agents and cytokines.

Ub is conjugated to target proteins through the conserved activity of E1 activating enzymes, E2 conjugating enzymes and E3 Ub ligases, and is reversed by deubiquitinases (DUBs) [1]. The Ub system comprises more than 800 genes encoding proteins that collectively modify more than 5000 cellular proteins, making ubiquitination one of the most widespread PTMs in the cell [1, 3, 4]. Ub is assembled into chains via any of seven internal lysine (Lys) residues or the N-terminal methionine (Met1), greatly increasing the complexity of Ub modifications [5]. The residue used to assemble Ub chains determines the chain topology and forms the basis for the pleiotropic cellular functions of Ub chains. For example, Lys48-linked Ub chains target proteins for proteasomal degradation whereas Lys63- and Met1-linked Ub chains are non-degradative and instead serve signalling functions by facilitating the assembly of protein complexes involved in immune signalling [2]. Less is so far known about the cellular function or the regulation of the other five Ub linkages [5].

Innate immune signalling is initiated by cytokine receptors such as TNF receptor 1 (TNFR1) and pattern recognition receptors (PRRs) such as Toll-like receptors (TLRs) and NOD-like receptors (NLRs) that recognise specific molecular structures of microbes [6]. When engaged by their cognate ligands, these receptors form multi-protein complexes where Lys63- and Met1-Ub signals are generated to stimulate various self-defence processes, including NF-κB-mediated inflammatory responses through activation of two Ub-dependent kinase complexes, the IκB Kinase (IKK) complex and the TGFβ-activated kinase 1 (TAK1) complex. Some innate immune receptors will also induce Met1-Ub-regulated signalling processes that restrict intracellular bacteria (e.g. NOD2) or instruct activation of cell death programmes (e.g. TNFR1 and TLR3) that contribute to restricting viral infections [7, 8]. The exquisite dependency of these cellular pathways on the generation of Met1-Ub signals is reflected by inflammatory diseases and immune deficiencies caused by inherited mutations and pathogen-encoded effector proteins affecting components of the Met1-Ub machinery [2, 9].

## The Met1-Ub machinery

Met1-Ub is assembled by the linear Ub chain assembly complex (LUBAC), the only known Ub ligase found in vertebrates to conjugate this Ub chain type, and is disassembled by the DUBs OTULIN and CYLD [10–12].

### Linear Ub chain assembly complex

**Fig. 1:**
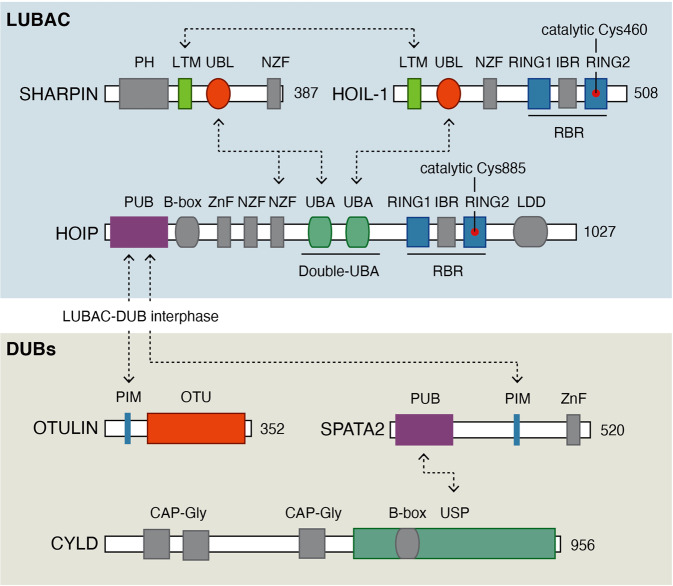
Schematic representation of the domain organisation of LUBAC subunits and associated DUBs. The catalytic cysteine in HOIP and HOIL are indicated with red circles and the numbering of all amino acid residues is based on the human protein. Abbreviations: B-box B-Box-type zinc finger, CAP-Gly cytoskeleton-associated protein-glycine rich, CBR catalytic in-between RING, IBR in-between-RING, CYLD cylindromatosis, HOIL-1 Heme-oxidised IRP2 Ub ligase-1, HOIP HOIL-1-interacting protein, LDD linear ubiquitin chains-determining domain, LTM LUBAC-tethering motif, LUBAC linear ubiquitin chain assembly complex, NZF nuclear protein localisation 4 (Npl4) zinc finger, OTU ovarian tumour, PH pleckstrin homology, PIM PUB-interacting motif, PUB peptide:N-glycanase/UBA- or UBX-containing proteins, RING really interesting new gene, SHARPIN Shank-associated RH domain-interacting protein, SPATA2 Spermatogenesis-associated 2, UBA ubiquitin associated, UBL ubiquitin-like, USP ubiquitin-specific protease, ZnF zinc finger. Arrows indicate domains that interact.

LUBAC is a trimeric complex composed of the subunits HOIL-1-interacting protein (HOIP; also termed RNF31, ZIBRA), Heme-oxidised IRP2 Ub ligase-1 (HOIL-1; also termed HOIL-1L, RBCK1, and RNF54), and Shank-associated RH domain-interacting protein (SHARPIN; also termed hSIPL1) [13–16] (Fig. 1). HOIP is the main catalytic component contributing to Met1-Ub formation by LUBAC although HOIL-1 in vitro is reported to have weak Met1-Ub ligase activity [17–20]. HOIP and HOIL-1 belong to the RING-in-between-RING (RBR) family of Ub ligases, which facilitate ubiquitination of substrates via the transfer of Ub from charged E2-Ub complexes via formation of a Ub-thioester intermediate with the catalytic cysteine in the RING2 in the RBR domain [17, 21]. On its own, HOIP is auto-inhibited through an intramolecular interaction between the RBR and the N-terminal part of the protein containing Ub-binding domains (UBDs) and an N-terminal peptide:N-glycanase/UBA- or UBX-containing proteins (PUB) domain [17]. HOIL-1 and SHARPIN harbour Ub-like (UBL) domains that interact with the UBDs in HOIP releasing the auto-inhibition of HOIP, and thereby forms the catalytic competent LUBAC complex [17, 18] (Fig. 1).

Although HOIL-1 has limited Ub ligase activity in vitro, recent studies indicate that it has previously unrecognised catalytic roles in LUBAC signalling [17, 22–24]. Fuseya et al. [23] showed that HOIL-1 conjugates monoUb to all LUBAC subunits and that this directs LUBAC to auto-ubiquitinate whilst attenuating the formation of Met1-Ub on receptor-interacting protein kinase 1 (RIPK1) following TNFR1 activation. This suggests that HOIL-1 not only contributes to release auto-inhibition of HOIP but also, through its Ub ligase activity, regulates the generation of Met1-Ub by LUBAC. Intriguingly, Kelsall et al. [24] reported that HOIL-1 conjugates Ub to serine and threonine residues through the generation of an oxy-ester bond with the C-terminus of Ub. In response to TLR activation, HOIL-1 facilitated ubiquitination of serine and threonine residues on components of the MyDDosome complex [24]. This suggests that HOIL-1 has a unique function as a serine/threonine Ub ligase in innate immune signalling albeit the functional role of these modifications remains to be explored. Nonetheless, it opens the possibility for additional Ub chain heterogeneity via oxy-ester bond ubiquitination as Ub itself contains several serine/threonine residues.

At a molecular level, HOIL-1 and SHARPIN individually release the auto-inhibition of HOIP but in cells HOIL-1 and SHARPIN are both necessary for LUBAC stability as ablation of SHARPIN or HOIL-1 results in substantially reduced levels of HOIP, resulting in strongly reduced or no LUBAC activity, respectively [14–17, 25, 26].

### LUBAC-associated DUBs

**Fig. 2:**
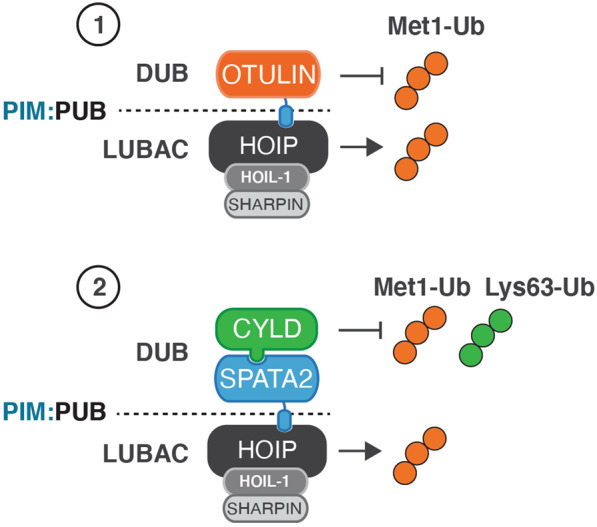
LUBAC–DUB complexes. LUBAC exists in distinct complexes with the DUBs OTULIN (1) and CYLD-SPATA2 (2). The LUBAC–DUB interactions are mediated by the PUB domain in HOIP and PUB-interacting motifs in OTULIN and SPATA2. The two LUBAC–DUB complexes can both generate Met1-Ub but have distinct DUB activities; OTULIN disassembles exclusively Met1-Ub whereas CYLD disassembles Met1-Ub and Lys63-Ub.

LUBAC-generated Met1-Ub levels are tightly regulated by the DUBs OTULIN and CYLD. OTULIN is an ovarian tumour protease (OTU)-family DUB that exclusively disassembles Met1-Ub [10, 27] and CYLD is a Ub-specific protease (USP)-type DUB that preferentially cleaves Met1-Ub and Lys63-Ub [28–30]. Both DUBs associate with LUBAC via interactions with HOIP to form LUBAC–DUB complexes [11, 12, 31]. The interaction is mediated by the PUB domain in the LUBAC subunit HOIP [12] and a conserved PUB-interacting motifs (PIMs) initially identified in OTULIN [32] (Fig. 1). The interaction with CYLD is mediated by the adaptor protein Spermatogenesis-associated 2 (SPATA2) [31, 33–35], which interacts with the CYLD USP domain and contains a PIM that facilitates binding to the HOIP PUB domain [31] (Fig. 1). OTULIN and SPATA2 bind the same PIM pocket in HOIP, which excludes the simultaneous binding of both proteins to LUBAC and gives rise to two distinct LUBAC–DUB complexes; LUBAC–OTULIN and LUBAC–SPATA2–CYLD [11, 31] (Fig. 2). The individual function of these complexes is not completely understood but since the LUBAC–SPATA2–CYLD complex in addition to regulation of Met1-Ub also disassembles Lys63-Ub it is likely that they have non-redundant roles in the regulation of LUBAC signalling (reviewed in [2]) (Fig. 2).

### Met1-Ub pathophysiology

Alongside the discovery and molecular characterisation of components of the Met1-Ub machinery, numerous genetic mouse models together with the identification of germline mutations in humans affecting the Met1-Ub machinery has greatly advanced our knowledge about the physiological and pathophysiological role of Met1-Ub: hypomorphic mutations in *HOIP* or truncating mutations in *HOIL-1* cause a complex clinical pathology characterised by autoinflammation, immunodeficiency and muscular amylopectinosis [36–38]. Germline mutations in *OTULIN* that destabilises OTULIN and/or interfere with its activity, cause an early onset autoinflammatory syndrome (termed OTULIN-related autoinflammatory syndrome) characterised by recurrent fevers, diarrhoea, panniculitis, autoantibodies and arthritis [39–42].

In mice, ablation of *Hoip*, *Hoil-1*, or *Otulin* leads to embryonic lethality [25–27, 43, 44] that involves aberrant cell death and signalling in part mediated by TNFR1. Cell type-specific deletion of *Otulin* in myeloid cells recapitulates many of the human OTULIN-mutation phenotypes whereas deletion of *Otulin* in B and T cells does not cause an overt phenotype [39]. No pathogenic germline human mutations have been described in *SHARPIN*, but in mice, a spontaneous frameshift mutation causes chronic proliferative dermatitis (*cpdm*), multiorgan organ inflammation and defects in the organisation of secondary lymphoid structures, and since HOIP and HOIL-1 levels are severely diminished, the *cpdm* mutant represents a LUBAC-depleted mouse [14–16, 45].

In humans, *CYLD* mono-allelic mutations underlie cutaneous tumour predisposition syndromes and somatic *CYLD* mutations have been linked to human papillomavirus-associated head and neck cancer [46, 47], whereas germline mutations in *SPATA2* have not been described.

## Met1-Ub in immune signalling

**Fig. 3:**
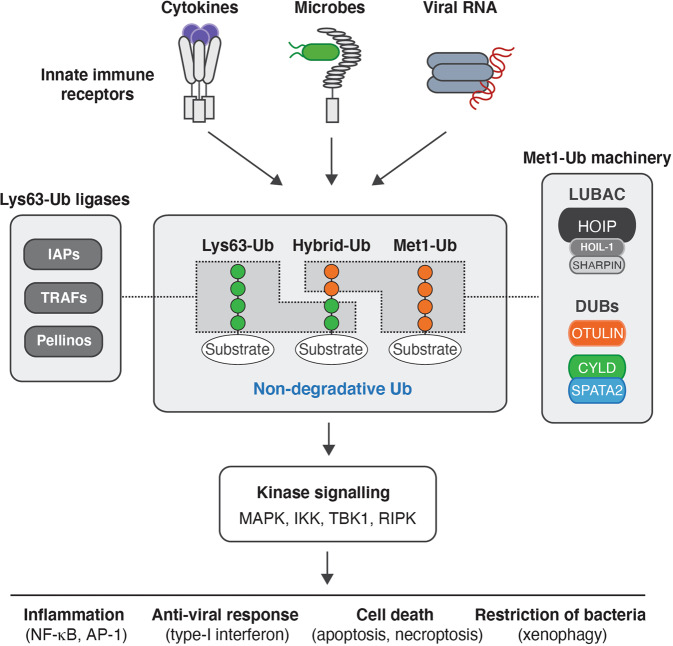
Ubiquitin in innate immune signalling responses. Schematic model showing the major ubiquitin chain types that control innate immune signalling responses. The Met1-Ub machinery (LUBAC, OTULIN, and CYLD-SPATA2) generates (and removes) Met1-Ub. Lys63-Ub is assembled by E3 Ub ligases such as Inhibitor of apoptosis (IAP) proteins, TNF-receptor associated factors (TRAFs), and Pellinos. Met1-Ub is conjugated to components of the receptor signalling pathway or to pre-existing Lys63-Ub to form hybrid Ub. These non-degradative Ub modifications serve as scaffolds for recruitment and activation of kinases that facilitate/regulate inflammation, anti-viral responses, cell death and xenophagy, depending on the nature of the activation signal(s).

A central step in innate immune receptor signalling is the recruitment of kinases belonging to the tyrosine-like kinase family; receptor-interacting protein kinases (RIPKs) and interleukin-1 receptor-interacting kinases (IRAKs). Here, these kinases serve as scaffolds for formation of Lys63-Ub conjugated by Inhibitor of apoptosis (IAP), TNF receptor-associated factor (TRAF), and Pellino protein family members (Fig. 3). The specific Ub ligase and the substrate(s) onto which it conjugates Lys63-Ub depends on the receptor engaged; RIPK1 is recruited to the TNFR1 signalling complex (termed complex I) and TLR3 where it is modified by Lys63-Ub by cIAP1/2 [48], RIPK2 is recruited to the intracellular bacteria-sensing receptors NOD1/2 where it is Lys63-Ub-modified by X-linked IAP (XIAP) and reportedly Pellino 3 [49, 50], IRAK1 and IRAK4 are recruited to TLR signalling complexes via the adaptor MyD88 where IRAK1/4 and MyD88 are modified by TRAF6 and Pellino1 [51]. The Lys63-Ub facilitates the recruitment and/or retention of LUBAC [49, 52], enabling the deposition of Met1-Ub on the existing Lys63-Ub as well as on other substrates in the complex [53, 54]. In turn, the Lys63-Ub and Met1-Ub promote the recruitment and activation of the TAB-TAK1 and NEMO-IKK kinase complexes through binding of TAB proteins to Lys63-Ub and NEMO to Met1-Ub, respectively, leading to activation of the transcription factor NF-κB and MAP kinase cascades and transcription of pro-inflammatory genes (Fig. 3). Lys63- and Met1-Ub also regulate anti-viral responses mediated by the kinases TBK1/IKKε and interferon response factors (IRFs), and cell-autonomous defence against intracellular bacteria [2]. In the context of TNFR1 or TLR3 signalling, Met1-Ub also functions independently of NF-κB signalling as an essential checkpoint to prevent the activation of RIPK1 kinase activity. Activated RIPK1 forms death-inducing complexes (termed complex II and necrosome) that induce caspase-8-dependent apoptosis or, under situations where caspase-8 is inhibited, RIPK3-dependent necroptosis (reviewed in [55, 56]).

LUBAC co-recruits SPATA2-CYLD to receptor signalling complexes where CYLD regulates Lys63-and Met1-Ub of substrates and thereby inflammatory and cell death signalling decisions [31, 33, 35, 57]. In contrast, OTULIN appears to be excluded from receptor signalling complexes [11, 31], but instead is essential to prevent LUBAC auto-ubiquitination and unrestricted accumulation of Met1-Ub [12, 23, 32, 39, 44, 58]. Despite not being stably present at receptor complexes, OTULIN restricts Met1-Ub at the NOD2 receptor complex to limit inflammatory signalling [58], suggesting that it can regulate LUBAC-mediated signalling independently of LUBAC-binding. In line with this, the majority of OTULIN is not associated with LUBAC [32], whereas the majority of CYLD seems to be associated with LUBAC [31]. The mechanisms regulating the interaction of LUBAC with OTULIN and SPATA2-CYLD in cells are not well-understood but in vitro studies show that phosphorylation of the tyrosine (Y56) in the OTULIN PIM abrogates its interaction with HOIP, suggesting that this may be a mechanism to regulate the LUBAC–OTULIN complex [32]. Supporting this notion, the phosphorylation of Y56 in OTULIN is reported to increase after cells were induced to undergo necroptosis (by treatment with TNF **+** cycloheximide+ the pan-caspase inhibitor zVAD-fmk), and, the phosphatase DUSP14 was found to counteract the phosphorylation [59]. Further investigations are needed to elucidate in more detail the consequence of the OTULIN phosphorylation and how it is regulated.

The OTU-family DUB A20 (encoded by *TNFAIP3*) disassembles Lys63- and Lys48-Ub and is essential for terminating inflammatory signalling. Intriguingly, A20 was recently found to be recruited to Met1-Ub at the TNFR1 signalling complex (and presumably other receptor signalling complexes) via its zinc finger seven (ZnF7), which selectively binds Met1-Ub. At the TNFR1, A20 stabilises Met1-Ub to inhibit cell death. Although A20 has DUB activity and Ub ligase activity, accumulating evidence indicate that its ability to interact with Met1-Ub via its ZnF7 domain is critical for its immune regulatory function [11, 60–62]. Thus, the three main DUBs that regulate immune receptor signalling, OTULIN, CYLD and A20, all regulate Met1-Ub directly or indirectly. How their activities are coordinated and regulated is not yet understood but will surely be elucidated in future studies.

## The Met1-Ub machinery in the host response to pathogens

Given that the Met1-Ub machinery regulates signalling processes by numerous PRRs it plays a central role of the host response to many viral and bacterial pathogens, which typically activate multiple receptors upon infection. In addition, Met1-Ub regulates signalling by pro-inflammatory cytokine receptors, notably TNFR1, and antigen receptors, linking this modification to amplification of PRR-induced responses and to the regulation of adaptive immune responses [63].

The direct involvement of LUBAC in viral immunity has been investigated in a number of recent studies. In response to infection by the RNA virus, influenza A virus (IAV), LUBAC-impaired *cpdm* mice are compromised in their host-defence response to a similar extent as mice deficient for the double-stranded RNA (dsRNA)-sensing receptor TLR3 [64]. Akin to *cpdm* mice, tissue-specific ablation of *Hoip* in alveolar epithelial cells led to increased lung injury and mortality of mice upon IAV infection in a manner that could be complemented by the introduction of constitutive active IKKβ [65]. Mechanistically, LUBAC is recruited to the TLR3 signalling complex where it conjugates Met1-Ub to stimulate NF-κB and IRF3 signalling, and cytokine production [64]. Consistently, small molecule HOIP inhibitors, HOIPINs, have been found to inhibit TLR3 signalling responses by the TLR3 ligand and the synthetic dsRNA analogue, poly (I:C) [66]. Curiously, ablation of *Hoil-1* in alveolar epithelial cells was shown to improve host-responses and increase survival to IAV infection relative to wild type mice [65]. It was proposed that this could be because the mice, due to the *Hoil-1* targeting of strategy (exon 8) [67], retained expression of a truncated N-terminal fragment of HOIL-1 containing the UBL and LTM and therefore retained LUBAC activity. Given that the truncated N-terminal fragment of HOIL-1 is similar to the recently described HOIL-1ΔRING1, which was shown to be unable to ubiquitinate HOIP and attenuate LUBAC activity [23], one might speculate that this mechanism underpins the improved response to IAV. Alternatively, the improved survival of the HOIL-1-targeted mice might represent an example where a ‘normal’ host-response to certain pathogens can be detrimental to the host. Irrespective, these studies suggest that LUBAC plays a key role in regulating optimal host-responses, balancing the risk of inducing cytokine storm versus the need to clear a pathogen, in this case, IAV [65].

LUBAC (HOIL-1) is also reported to mediate type-I and -III IFN responses and the control of viral titters in vivo following murine norovirus infection, an RNA virus sensed by the cytosolic RIG-I-like receptor melanoma differentiation-associated protein 5 (MDA5) [68]. In contrast to its role in MDA5 signalling, LUBAC is reported to either suppress or to be dispensable for the RIG-I mediated type-I IFN and NF-κB responses following vesicular stomatitis virus (VSV) and Sendai virus (SeV) [68–70]. LUBAC negatively regulates the RIG-I pathway by counteracting the Lys63-Ub ligase TRIM25, which is essential for RIG-I signalling [69, 71], but its molecular role in MDA5 signalling is not known. In addition to suppression of RIG-I signalling, LUBAC was recently reported to negatively regulate VSV-induced IFN responses through ubiquitination of the transcription factor STAT1, and thereby to interfere with the interaction of STAT1 with the type-I IFN receptor IFNAR2 [72]. Mice heterozygous for HOIL-1 deletion displayed enhanced IFN-β levels and a reduced viral load following VSV infection. This suggests that Met1-Ub may be a general regulator of STAT1-dependent expression of type-I IFN induced genes.

Along this line, ablation of *Hoil-1* in the context of *Ripk3*^*−*/*−*^*Casp8*^−/−^ results in accumulation of IFN-inducible chemokines CXCL10 and CXCL9 in mouse embryos compared to *Ripk3*^*−*/*−*^*Casp8*^*−/−*^ embryos [25]. Elevated levels of type-I IFN was also recently reported in mice with liver parenchymal cell-specific ablation of *Otulin* and this was found to be dependent on the type-I IFN receptor IFNAR1 [73]. A separate study found that expression of catalytic inactive OTULIN (C129A) in the context of *Ripk3*^*−*/*−*^*Casp8*^−/−^ leads to accumulation of the IFN-inducible chemokines CXCL10 and CXCL9, which was suppressed by blocking IFNAR1 with an antibody [44]. Interestingly, the deletion of one allele of *Ripk1* rescued the lethality of *Otulin*^*C129A/C129A*^ *Ripk3*^*−*/*−*^*Casp8*^−/−^ mice and reduced the level of IFN-inducible chemokines [44]. Akin to this, ablation of *Ripk1* normalised levels of type-I IFN and CXCL-10 and rescued the lethality of *Hoil-1*^*−*/*−*^ *Ripk3*^*−*/*−*^*Casp8*^−/−^ mice [25], suggesting a key role for RIPK1 in mediating IFN responses when the Met1-Ub machinery is perturbed and cell death is inhibited. Whereas deletion of HOIL-1 directly interferes with LUBAC activity, resulting in reduced levels of Met1-Ub, the absence of OTULIN or its activity results in extensive accumulation of Met1-Ub. A proposed model for why these two scenarios result in a similar outcome is that LUBAC, in the absence of OTULIN (or its activity) leads to LUBAC autoubiquitination, which in turn interferes with its function in receptor signalling. However, this model is not obviously reconcilable with studies demonstrating that OTULIN-deficiency leads to excessive LUBAC-dependent pro-inflammatory signalling [10, 39, 58, 74].

While LUBAC prevents aberrant IFN signalling, it is necessary for efficient NF-κB activation in response to bacterial sensing. In addition to its role in mediating NF-κB activation downstream of bacteria-sensing PRRs [15, 49, 58], LUBAC also ‘labels’ intracellular bacteria such as *Salmonella typhimurium* with Met1-Ub [74, 75]. The bacteria-associated Met1-Ub functions as a recruitment platform for assembly of a signalling complex to initiate innate immune signalling via NF-κB. The Met1-Ub additionally targets the bacterium for xenophagy, a process for selective autophagy of cytosolic bacteria and viruses which helps eliminate pathogen infection [74–76]. HOIP-mediated Met1-Ub recruits autophagy receptors such as Optineurin [75], which like NEMO selectively binds Met1-Ub and thereby links the autophagy machinery to the Met1-Ub to restrict the proliferation of cytosolic *Salmonella typhimurium* [74, 75]. OTULIN also regulates Met1-Ub on the cytosolic bacteria and regulates NF-κB activation by disassembling Met1-Ub associated with the bacteria [74]. The Met1-Ub machinery is also reported to stimulate xenophagy of cytosolic *S. typhimurium* through Met1-Ub modification of ATG13, a component of the ULK1 initiation complex [77, 78]. ATG13 was found to interact with OTULIN and depletion of HOIP decreased autophagy and increased replication of *S. typhimurium* [77].

CYLD has been implicated as a negative regulator of the host response to pathogens, including non-typeable *Haemophilus influenza* [79], *Escherichia coli* [80], *Listeria monocytogenes* [81], *Streptococcus pneumoniae* [82], cerebral malaria [83]. CYLD-deficient mice display an exacerbated inflammatory response to pathogen infection, which, depending on the pathogen and/or experimental system is beneficial or detrimental to the host. For example, CYLD-deficient mice have an increased capacity to clear *Listeria monocytogenes* infection as compared to wild type mice, in part is due to the increased ability of macrophages to restrict the intracellular accumulation of the bacterium through a mechanism involving the NOD1/2 adaptor kinase RIPK2 [81, 84]. Conversely, CYLD-deficient mice were recently found to be susceptible to *Citrobacter rodentium* infection due to overactivation of NLRP6 and IL-1β production of CYLD-deficient macrophages [85]. However, it remains uncertain if CYLD exerts its effect through regulation of K63-Ub, Met1-Ub or a combination of both.

## Pathogen effectors targeting the Met1-Ub machinery

**Fig. 4:**
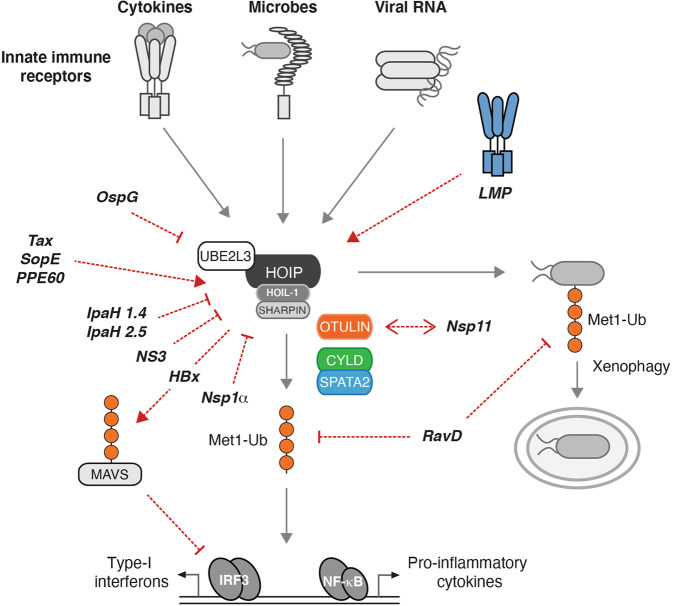
Pathogen effectors targeting the Met1-Ub machinery. Schematic model of the major Met1-Ub-controlled pathways activated by pathogens, and of the bacterial and viral effectors that modulate the Met1-Ub machinery. Activation of receptors by cytokines or molecular patterns from microbes leads to the formation of Met1-Ub and ultimately transmission of signals from the activated receptor to initiate anti-bacterial autophagy or transcription factor activation via nuclear factor-κB (NF-κB) and interferon response factor 3 (IRF3). Red arrows and lines denote where in the pathway the effectors exert their function. The pathogen effectors depicted target Met1-Ub in various direct or indirect ways. Abbreviations: HBx hepatitis B virus X protein, IpaH1.4 invasion plasmid antigen H1.4, IpaH2.5 invasion plasmid antigen H2.5, IRF7 interferon regulatory factor 7, LMP1 latent membrane protein 1, MAVS mitochondrial antiviral-signalling protein, NS3 non-structural protein 3, NSP1α non-structural protein 1α, Nsp11 non-structural protein 11, OspG outer Shigella protein G, PPE60 proline–proline–glutamate motifs 60, RavD region allowing vacuole colocalization D, SopE Salmonella outer protein E, Tax trans-activator protein X.

To advance the capacity to successfully infect their host, pathogens have evolved a wide range of effector molecules that specifically target host proteins or pathways needed for mounting an efficient host-defence. Given its role in innate immune regulation and cell-intrinsic host defence processes, it is not surprising that the Met1-Ub machinery is targeted both indirectly and directly by viral and bacterial proteins. Subversion of Met1-Ub by effectors appear to either antagonise or co-opt LUBAC, depending on the type of pathogen (summarised in Fig. 4 and Table 1).

**Table 1:** Pathogen effectors targeting the Met1-Ub machinery.

Pathogen	Effector	Protein type	Mechanism
*Bacteria*			
*Shigella flexneri*	IpaH1.4 IpaH2.5	E3 Ub ligase	Lys48 ubiquitination of HOIP followed by proteasomal degradation [97]
	OspG	Kinase	Sequestering the E2 UBE2L3 [97–99]
*Legionella pneumophila*	RavD	Deubiquitinating enzyme	Cleaves Met1-Ub specifically [106]
*Salmonella typhimurium*	SopE	Guanine nucleotide exchange factor	Activates LUBAC [100]
*Mycobacterium tuberculosis*	PPE60	Proline-proline-glutamate (PPE) motifs	Activates LUBAC [101]
*Viruses*			
Hepatitis C virus (HCV)	NS3	Serine protease and RNA helicase	Interacts with HOIP and prevents NF-κB signalling [86]
Porcine reproductive and respiratory syndrome virus (PRRSV)	Nsp1α	Protease	Disruption of HOIP interaction with SHARPIN [87]
	Nsp11	Non-structural protein	Interacts with OTULIN and inhibits NF-κB signalling [88]
Epstein-Barr virus (EBV)	LMP1	TNFR superfamily	Recruits LUBAC to ubiquitinate and inactivate IRF7. Averts anti-viral interferon production [92]
			Recruits LUBAC to activate NF-κB oncogenic signalling [91]
Human T cell leukaemia virus type 1 (HTLV-1)	Tax	Trans-activator protein	Recruits LUBAC to activate NF-κB signalling in leukemogenesis [94]
Hepatitis B virus (HBV)	HBx	Trans-activator protein	Blocks MAVS signalling by recruiting Parkin-associated LUBAC [93]

Abbreviations: *HBx* hepatitis B virus X protein, *IpaH1.4* invasion plasmid antigen H1.4, *IpaH2.5* invasion plasmid antigen H2.5, *IRF7* interferon regulatory factor 7, *LMP1* latent membrane protein 1, *MAVS* mitochondrial antiviral-signalling protein, *NS3* non-structural protein 3, *NSP1* non-structural protein 1, *Nsp11* non-structural protein 11, *OspG* outer Shigella protein G, *PPE60* proline–proline–glutamate motifs 60, *RavD* region allowing vacuole colocalization D, *SopE* Salmonella outer protein E, *Tax* trans-activator protein X.

### Viral effectors

Hepatitis C virus, a single-stranded RNA virus, expresses ten viral proteins including a serine protease, NS3. NS3 binds the NZF2 domain in HOIP (Fig. 1) and competes for NEMO-binding in vitro. In cells, NS3 can antagonise TNF-induced NF-κB and the recruitment of NEMO to Met1-Ub [86]. The porcine reproductive and respiratory syndrome virus (PRRSV) expresses two effectors that have been found to target the Met1-Ub machinery, Nsp1α and Nsp11. Nsp1α interferes directly with LUBAC by antagonising the SHARPIN–HOIP interaction, which reduces the recruitment of NEMO to Met1-Ub in cells infected with PRRSV and treated with TNF [87]. Nsp11 was reported to interact with OTULIN and the IKK complex, and to inhibit NF-κB activation in response to SeV infection but the mechanism is unclear [88].

Some viral effectors recruit LUBAC to decorate host factors with Met1-Ub either impeding on their function by blocking their interaction partners or driving NF-κB signalling which ultimately can transform cells. The oncogenic gammaherpesvirus Epstein Barr Virus (EBV) transforms human primary B-cells, which in vitro is dependent on EBV nuclear antigens together with latent membrane proteins (LMPs) [89]. LMP1 is a member of the TNFR superfamily, which independently of ligand-binding stimulates constitutive NF-κB activity and IRF7-mediated type-I IFN responses [90]. LMP1 recruits LUBAC via interaction with HOIP, which stimulates activation of NF-κB and represses type-I IFN signalling, suggestively by directing LUBAC to ubiquitinate TRAF1/2, NEMO and IRF7 [91, 92]. Similarly, hepatitis B virus (HBV) employs the protein HBx to direct LUBAC to inhibit an antiviral response by conjugating Met1-Ub to mitochondrial antiviral signalling (MAVS) protein. The ubiquitination of MAVS inhibits assembly of the MAVS signalosome and impaired IRF3-mediated type I interferon [93]. Human T cell leukaemia virus type 1 (HTLV) is a retrovirus associated with adult T-cell leukaemia. Using cell extracts from Jurkat cells and the HLTV protein Tax, Shibata et al. show that Tax coordinates assembly of a signalling complex where LUBAC together with an unknown Lys63-Ub ligase generates hybrid Lys63-Met1-Ub which activates IKK and NF-κB, which is suggested to stimulate proliferation of HLTV infected cells [94].

### Bacterial effectors

The Ub machinery is not present in prokaryotes or viruses, but many pathogens have evolved E3 ligase and DUB-like proteins [95] and these are part of a range of effector proteins that are injected into host cells using bacterial secretion systems. Many effectors target the Ub machinery in one way of the other, and some target LUBAC Met1-Ub specifically.

Recently a bacterial effector that targets Met1-Ub specifically was identified in *Legionella pneumophila*, an intracellular bacteria and the causative agent of human Legionnaires’ disease [96]. The DUB activity was identified through a screen for Met1-Ub activity using lysates from 43 bacterial species [96]. Out of ~300 *Legionella* effector proteins, RavD was identified as the causative effector protein that could degrade Met1-Ub and inhibit LUBAC-mediated NF-κB activation by TNF. Structural analysis revealed that RavD was specific to Met1-Ub and has evolved a similar Met1-Ub interaction-surface as OTULIN [96]. RavD deletion did not abolish *Legionella* ability to replicate in macrophages but caused increased NF-κB activation upon infection, suggesting it contributes to host immune suppression [96]. A RavD orthologue was identified in *Legionella clemsonensis* and Met1-Ub DUB activity was detected in lysates from *Legionella bozemanni*, suggesting Met1-Ub DUB activity is a general mechanism for *Legionella* species [96].

Other bacteria encode effectors that target Met1-Ub through other mechanisms. The enteroinvasive bacteria, *Shigella flexneri*, encodes the Ub ligases IpaH1.4 and IpaH2.5 that modify HOIP with K48-Ub, marking it for proteasomal degradation [97]. IpaH1.4 has also been shown to antagonise Met1-Ub on cytosolic *Shigella*, which limited recruitment of the xenophagy machinery [75]. In addition, *Shigella* OspG is thought to inhibit LUBAC activity indirectly through sequestration of the UBE2L3, which LUBAC uses for Met1-Ub assembly [97–99]. Instead of inhibiting LUBAC, the *Salmonella typhimurium* effector SopE was found to stimulate LUBAC activity [100]. SopE is a guanine nucleotide exchange factor (GEF) and contributes to the remodelling of the host-cell actin cytoskeleton rearrangement upon infection. Ectopic expression of SopE activates LUBAC and a *Salmonella typhimurium* mutant deleted for SopE and two closely related GEFs induced less Met1-Ub upon infection than its wild type counterpart [100]. However, whether the activation of LUBAC contributes to the pathogenicity or if it is a side-effect of SopE’s remodelling of the cytoskeleton remains to be determined. There are indications that *Mycobacterium tuberculosis* also induces LUBAC activity via effector protein PPE60 although the mechanism remains unknown [101]. In the study, the fungal metabolite gliotoxin was used to inhibit LUBAC. Gliotoxin binds HOIP and can inhibit its function [102]. However, a subsequent study reported that gliotoxin is cytotoxic and did not inhibit NF-κB activation at sublethal concentrations, which raises questions about the specificity of gliotoxin as an effector targeting LUBAC [66].

## Conclusions and outlook

In the 15 years since the discovery of the first components of the Met1-Ub machinery, HOIP and HOIL-1, tremendous progress has been made towards understanding of the biological function of Met1-Ub and of the cellular machinery that generates, binds, and disassembles this PTM. It is now evident that Met1-Ub plays crucial roles in the regulation of inflammatory signalling and cell death by innate immune receptors. As inappropriate regulation of Met1-Ub gives rise to severe and potentially fatal pathologies, the enzymes that generate and remove Met1-Ub must be tightly regulated. The identification of DUBs that cleave Met1-Ub, OTULIN and CYLD, and the realisation that these exist in stable complexes with LUBAC, provided the first insights into the regulation of LUBAC. However, it remains less clear what the role of the individual LUBAC–DUB complexes is and how they are regulated. It will also be important to further elucidate how LUBAC itself is regulated, for example by different PTMs. The recent discovery that HOIL-1 ubiquitinates LUBAC to regulate its activity together with the finding that HOIL-1 conjugates Ub onto serine/threonine residues is intriguing and warrants further investigation. The accumulating evidence showing an important role for Met1-Ub also in regulating antiviral IFN responses and xenophagy, underscores the complex functions of this PTM in coordinating immune responses and cell-autonomous host defence strategies. It is therefore not surprising that both viruses and bacteria encode effectors that target the Met1-Ub machinery. While some have been described in detail, others still require further characterisation in order to fully understand their mechanism of action, and many are probably yet to be discovered. A better understanding of the interplay between the Met1-Ub machinery and pathogen effectors might indeed offer insight into how the Met1-Ub machinery is regulated, which ultimately may reveal ‘druggable’ targets. Targeting the Ub system offers exiting therapeutic possibilities to modulate inflammation and cancer [103] and small-molecules to inhibit the catalytic activity of HOIP have recently been described [102, 104, 105]. A potential therapeutic benefit remains to be explored, but one can imagine that LUBAC inhibitors could alleviate hyper inflammation conditions either in chronic conditions or in acute infections with viruses such as IAV [65] and SARS-CoV-2.
